# RICH1 inhibits breast cancer stem cell traits through activating kinases cascade of Hippo signaling by competing with Merlin for binding to Amot-p80

**DOI:** 10.1038/s41419-022-04516-2

**Published:** 2022-01-21

**Authors:** Qi Tian, Huan Gao, Yan Zhou, Lizhe Zhu, Jiao Yang, Bo Wang, Peijun Liu, Jin Yang

**Affiliations:** 1 Department of Medical Oncology, The First Affiliated Hospital of Xi’an Jiaotong University, Xi’an, China; 2 Department of Breast Surgery, The First Affiliated Hospital of Xi’an Jiaotong University, Xi’an, China; 3 Center for Translational Medicine, The First Affiliated Hospital of Xi’an Jiaotong University, Xi’an, China

**Keywords:** Breast cancer, Cancer stem cells

## Abstract

Cancer stem cells (CSCs) are regarded as the root of tumor recurrence and distant metastasis, as well as the major cause of resistance to conventional cancer therapies. Elucidating the mechanism of regulating CSCs is of great significance for the development of CSCs-targeting therapy strategies. YAP/TAZ are identified as key regulators of CSCs-related traits on breast cancer cells; however, the upstream regulatory mechanism of Hippo kinases cascade involved in regulating YAP/TAZ remains elusive. In this study, we found that the low expression of RICH1 in breast cancer was associated with poor prognosis. Depletion of RICH1 promoted the stemness and disrupted the normal epithelial architecture of MCF10A cells. Besides, RICH1 inhibited the migration and invasion of breast cancer cells and sensitized these cells to chemotherapeutic drugs. Mechanistically, RICH1 activated the kinases cascade of Hippo signaling via displacing Amot-p80 from the complex with Merlin. Further studies revealed that the deletion of the BAR domain of RICH1 abolished the function of attenuating the binding of Amot-p80 and Merlin, illustrating that the competitive binding to Amot-p80 with Merlin was mediated by the BAR domain of RICH1. In conclusion, our work elucidated the role and molecular mechanism of RICH1 in stemness regulation of breast cancer, and might provide opportunities for CSCs-targeting therapy.

## Introduction

Breast cancer is the most common cancer in the world [1]. Although the clinical application of a variety of new therapeutic regimens has significantly improved the prognosis of breast cancer patients, there are still a majority of patients inevitably suffering from disease recurrence or metastasis. Cancer stem cells (CSCs), the subpopulation of tumor cells with infinite self-renewal capacity, are considered as the root of recurrence and metastasis, as well as the major cause of resistance to conventional cancer therapies [2]. Although several studies have revealed the regulatory mechanism of breast cancer stem-like cells (BCSCs) [3–5], our understanding of BCSCs is still very limited. Elucidating the mechanism of regulating BCSCs is of great significance for the development of CSCs-targeting therapy strategies.

The paralogous transcriptional coactivators YAP/TAZ of the Hippo pathway have been identified as key regulators of CSCs-related traits on breast cancer cells [6–9]. The tumor suppressor gene *NF2* encodes a 69 kDa protein called Merlin, which involves in the canonical Merlin/*NF2*-Mst1/2-Lats1/2-YAP/TAZ axis, initiating the Hippo signaling by directly activating Mst1/2, or by recruiting Lats1/2 to membrane for phosphorylation by Mst1/2 [refs. 10–12]. Although the role of Merlin in regulating the Hippo kinases cascade has been well studied, the upstream regulators of Merlin in the Hippo pathway remain elusive.

RICH1, also known as ARHGAP17 (Rho GTPase Activating Protein 17), belongs to the GTPase-activating proteins (GAP) family, and its function of catalyzing the GTP hydrolysis of small G proteins of Rho family has been fully investigated [13]. It was originally identified in neurocytes, and its deletion was found to induce cytoskeleton remodeling in nerve terminations and promote Ca^2+^-dependent exocytosis by activating intrinsic enzymatic activities of Rac1 and Cdc42 [ref. 14]. RICH1 can also form a complex with ERM protein, specifically inhibiting RhoA to promote the morphological differentiation of astrokeratinocytes into astrocytes [15]. In platelets, RICH1 was found to inhibit the formation of stress fibers and pseudopods by regulating the GTPases activity of Cdc42, Rac1, or RhoA, and ultimately platelet adhesion and aggregation were significantly reduced [16–18]. It was also suggested that RICH1 could inhibit the formation of filopodia in breast cancer cells [19], indicating the potential tumor suppressor function of RICH1. However, the mechanisms underlying the role of RICH1 in the regulation of tumor progression remain largely unknown.

RICH1 is mainly composed of three functional domains, an N-terminal BAR domain, a RhoGAP domain, and a C-terminal tail with multiple proline-rich motifs. In addition to regulating Rho signaling, RICH1 is also involved in the formation of tight junctions and maintenance of apical-basal polarity of epithelial cells, by anchoring to the Crumbs complex (composed of CRB, PALS1, and PATJ) and the PAR complex (composed of PAR3, PAR6, and aPKC), through binding to the scaffold protein Amot by the BAR domain. Moreover, the close relationships between RICH1 and other apical-basal polarity-regulating molecules upstream of the Hippo pathway have been reported in several previous studies. For example, Wells et al. [13] reported that the RICH1/Amot complex was involved in the maintenance of apical-basal polarity of epithelial cells MDCK by regulating the GTPase activity of Cdc42; Yi et al. [20] also confirmed that the tight junction-associated Merlin-Amot complex regulated the mitogenic signaling by modulating the hydrolytic activity of RICH1. While Amot and Merlin are well-recognized as upstream regulators of the Hippo pathway, whether RICH1 can regulate the Hippo signaling has not been reported.

In the present study, we revealed that the low expression of RICH1 is associated with poor prognosis and CSCs-like properties in breast cancer. RICH1 overexpression could activate the kinases cascade of Hippo signaling by competing with Merlin for binding to Amot-p80, which further inhibits stemness and improve the chemosensitivity of breast cancer cells.

## Results

### Low expression of RICH1 is associated with poor prognosis in breast cancer patients

**Fig. 1:**
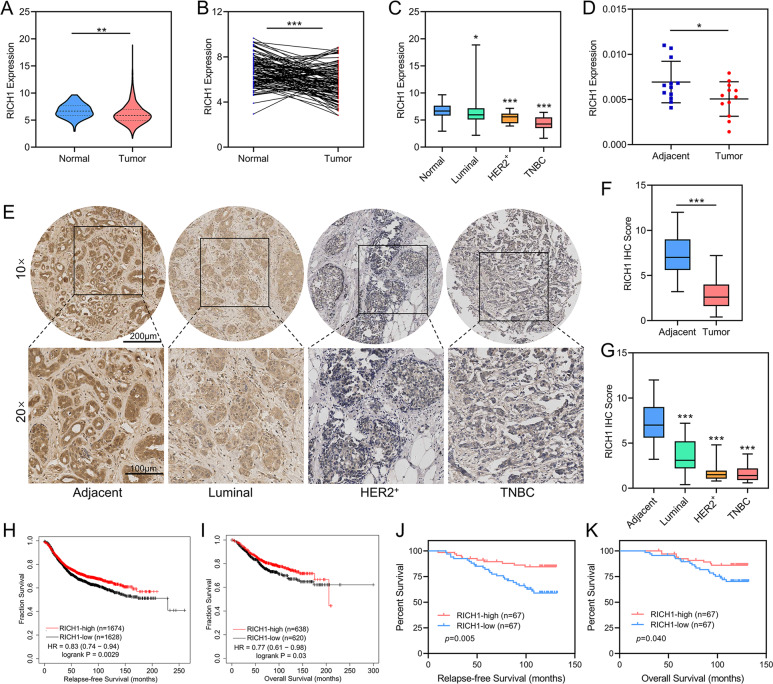
Low expression of RICH1 is associated with poor prognosis in breast cancer patients. **A** Violin plot of RICH1 mRNA expression in breast cancer and normal mammary tissues obtained from TCGA database. **B** RICH1 mRNA expression levels in paired breast cancer compared to matched normal tissues obtained from TCGA database. **C** Box plot of RICH1 mRNA expression in different subtypes of breast cancer and normal tissues obtained from TCGA database. **D** The mRNA expression of RICH1 in 12 pairs of fresh postoperative breast cancer and adjacent mammary tissues. **E** Representative images of IHC staining results of RICH1 in breast cancer tissue microarray. **F** Box plot showing the protein expression levels of RICH1 in 135 breast cancer and 57 adjacent breast tissues. **G** Box plot showing the protein expression levels of RICH1 in different subtypes of breast cancer and adjacent mammary tissues. **H**, **I** Kaplan-Meier survival analysis of RFS and OS of breast cancer patients stratified by RICH1 expression levels according to the online Kaplan-Meier plotter website. **J**, **K** Kaplan-Meier survival analysis of RFS and OS of breast cancer patients separated by the median value of RICH1 IHC score. *P* values in **A**, **C**, **D**, **F**, and **G** were calculated using unpaired two-tailed Student’s *t* tests. *P* value in **B** was calculated using paired two-tailed Student’s *t* tests. *P* values in **H**–**K** were calculated using log-rank text. Abbreviation: TCGA the Cancer Genome Atlas, IHC immunohistochemistry, RFS relapse-free survival, OS overall survival; *p < 0.05; **p < 0.01; ***p < 0.001.

To investigate the role of RICH1 in breast cancer, we downloaded the RNA-seq data of RICH1 from 1104 breast cancer samples and 113 normal breast tissues from the TCGA database and found that RICH1 was significantly decreased in breast tumors (Fig. 1A). RICH1 was also low expressed in 110 paired breast tumor tissues compared to matched normal tissues (Fig. 1B). In addition, the expression levels of RICH1 in HER2-positive and TNBC samples were significantly lower than in luminal subtypes (Fig. 1C). The low expression of RICH1 mRNA in breast cancer was also validated by 12 pairs of fresh postoperative breast cancer and adjacent tissues (Fig. 1D). We further performed immunohistochemistry (IHC) staining with commercial tissue microarrays (HBreD140Su03 and HBreD077Su01) and found that RICH1 was more highly expressed in noncancerous tissue regions and weakly detected in tumor tissues, especially in tumors with high degree of malignancy, such as HER2-positive and TNBC samples (Fig. 1E–G).

An online Kaplan-Meier plotter breast cancer survival analysis was applied to assess the clinical significance of RICH1 in predicting clinical outcomes (www.kmplot.com). The results revealed that tumors with higher RICH1 mRNA expression had significantly increased relapse-free survival (RFS, Fig. 1H) and overall survival (OS, Fig. 1I). We then investigated whether RICH1 protein expression was associated with prognosis in 134 breast cancer patients. Patients were separated into two groups using the median value of RICH1 IHC score as the dividing line and Kaplan-Meier survival analysis was performed to show high RICH1 expression positively correlated with increased RFS (Fig. 1J) and OS (Fig. 1K). Further correlation analysis in Table 1 showed that, tumors with low RICH1 expression had higher propensity for lymph node metastasis and higher pathological grade and clinical stage. In addition, higher negative rates of ER and PR, and more TNBC samples were observed in tumors with low RICH1 expression.

**Table 1:** Correlation analysis of RICH1 expression levels and clinicopathological parameters in breast cancer patients.

Characteristics	Total	RICH1-low	RICH1-high	*p* value
Age (y)				0.21
≤60	94	50	44	
> 60	41	17	24	
T				0.421
T1	55	25	30	
T2-T3	80	42	38	
N				<0.001
N0	71	25	46	
N1-N3	64	42	22	
Pathological grade				0.022
II	93	40	53	
III	42	27	15	
AJCC stage				<0.001
I-II	90	30	60	
III	45	37	8	
ER status				0.005
negative	40	27	13	
positive	91	37	54	
PR status				0.015
negative	61	37	24	
positive	71	28	43	
Her2 status				0.367
negative	108	52	56	
positive	24	14	10	
TNBC				0.01
no	104	45	59	
yes	25	18	7	

### Low expression of RICH1 is associated with the enhancement of CSC properties in breast cancer

**Fig. 2:**
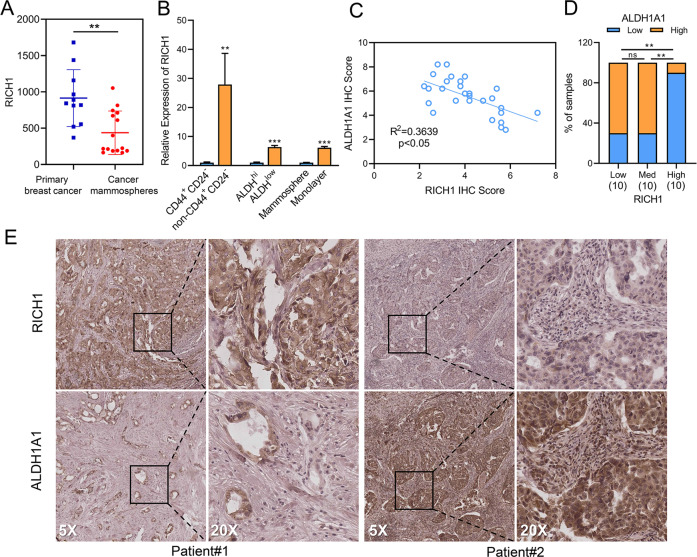
Low expression of RICH1 is associated with the enhancement of CSC properties in breast cancer. **A** RICH1 expression in primary bulk tumor samples and mammospheres provided by GSE7515. **B** RICH1 mRNA expression levels in BCSC-like subpopulations (CD44^+^CD24^**−**^, ALDH^hi^ and mammospheres) and non-BCSC-like subpopulations (non-CD44^+^CD24^**−**^, ALDH^low^ and adherent monolayer). **C** Correlation analysis between RICH1 and ALDH1A1 intensities in 30 breast cancer samples. **D** ALDH1A1 expression levels in breast cancer samples with different RICH1 expression. **E** Representative IHC images of RICH1 and ALDH1A1 of 2 breast cancer patients, PATIENT#1 (case number of #1525760) and PATIENT#2 (case number of #1392301). Among them, PATIENT#1 represented an example with high expression of RICH1 and low expression of ALDH1A1, while PATIENT#2 represented another example with low expression of RICH1 and high expression of ALDH1A1. Data represent mean ± SD. Statistical significance was determined by two-tailed unpaired *t*-test (**A**, **B**), pearson’s r (**C**) or chi-squared test (**D**). Abbreviation: CSC cancer stem cell, BCSC breast cancer stem cell, ns not significant; *p < 0.05; **p < 0.01; ***p < 0.001.

In search of reasons that RICH1 influencing the prognosis of breast cancer patients, we interrogated GSE7515, a gene set including expression profiles taken from 11 primary bulk tumor samples and 15 mammosphere samples derived from human breast tumors [21], and found that RICH1 was significantly down-regulated in cancer mammospheres (Fig. 2A). We then sorted BT549, a human TNBC cell line, into BCSC-like subpopulations (CD44^+^CD24^-^ and ALDH^hi^) and non-BCSC-like subpopulations (non-CD44^+^CD24^-^ and ALDH^low^) via flow cytometry, and found the mRNA expression level of RICH1 was much higher in non-BCSC-like subpopulations (Fig. 2B). In addition, we analyzed RICH1 mRNA levels in BT549 cells, which were cultured as adherent monolayers or nonadherent spheroids, and the results showed that RICH1 mRNA level was decreased in mammosphere cultures (Fig. 2B). To ensure the clinical relevance of BCSC-associated marker and RICH1, we assessed the expression of RICH1 and ALDH1A1 by IHC in 30 breast cancer samples. A negative correlation between RICH1 and ALDH1A1 intensities was found in these samples (Fig. 2C, E). Meanwhile, the samples were divided into three groups according to the RICH1 IHC scores, and significantly stronger ALDH1A1 expression was found in tumors with lower RICH1 expression (Fig. 2D). The above results suggested that RICH1 might play a negative regulatory role in BCSC maintenance.

### RICH1 inhibits stem cell-like properties in breast cancer and maintains the normal epithelial architecture of MCF10A cells

**Fig. 3:**
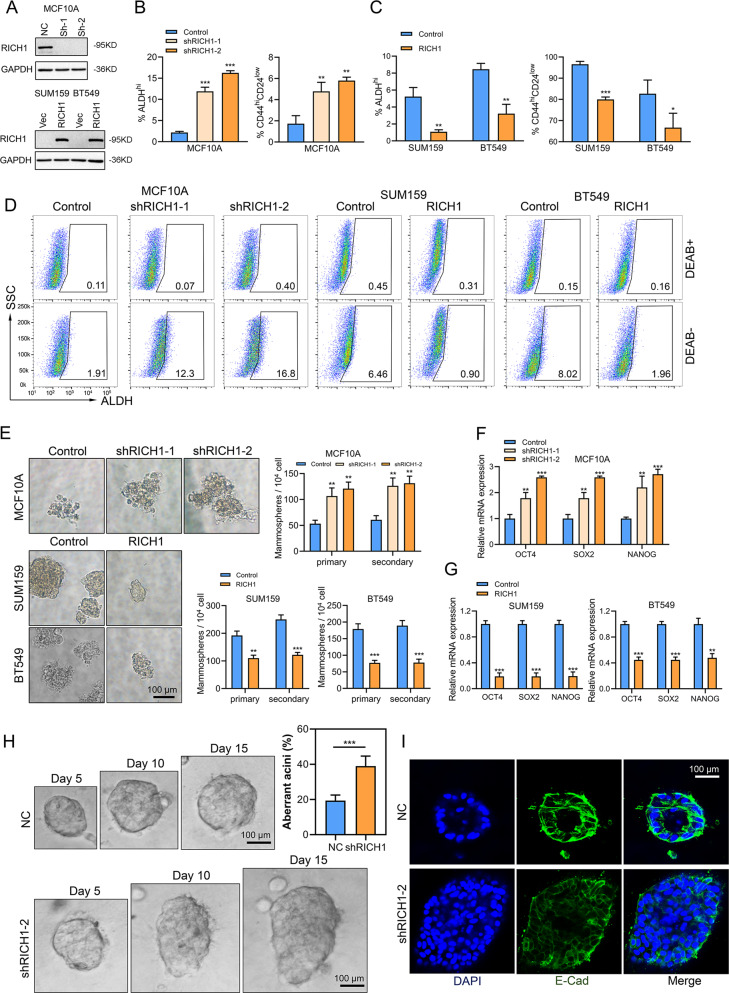
RICH1 inhibits stem cell-like properties in breast cancer and maintains the normal epithelial architecture of MCF10A cells. **A** RICH1 knockdown in MCF10A and overexpression in SUM159 and BT549 cells. **B**–**D** Flow cytometry analysis and representative images of ALDH^hi^ and CD44^+^CD24^-^ subpopulations in MCF10A, SUM159, and BT549 cells. Numbers in the flow cytometry charts indicate the CSC percentages. **E** Quantitation and representative images of primary and secondary tumorsphere formation in RICH1-knockdown MCF10A and RICH-overexpressing breast cancer cells. **F**, **G**. Relative mRNA levels of CSC-TFs (OCT4, SOX2, and NANOG) after RICH1 knockdown (**F**) and overexpression (**G**). **H** Morphogenesis and aberrant acini formation rate of MCF10A cells plated on matrigel 3D culture. **I** NC- or shRICH1-transfected MCF10A cells in matrigel 3D culture stained with E-cadherin on day 15. All the experiments were repeated three times independently with similar results, and the data of one representative experiment are shown. Data represent mean ± SD. Statistical significance was determined by two-tailed unpaired *t*-test (**B**, **C**, **E**–**H**). Abbreviation: CSC-TFs CSC properties-associated transcription factors; *p < 0.05; **p < 0.01; ***p < 0.001.

To investigate the role of RICH1 in BCSCs, breast epithelial cell MCF10A with RICH1 knockdown (MCF10A-shRICH1-1 and MCF10A-shRICH1-2), and breast cancer cells SUM159 and BT549 with RICH1 overexpression (SUM159-RICH1 and BT549-RICH1) were established using RICH1-targeted lentivirus (Fig. 3A), followed by flow cytometry analysis of CSC contents in these cell lines. RICH1 knockdown led to CD44^+^CD24^-^ and ALDH^hi^ cell subpopulations expansion in MCF10A, whereas the proportions of CD44^+^CD24^-^ and ALDH^hi^ subpopulations were decreased in RICH1 overexpression cells (Fig. 3B–D and Supplementary Figure 1). The EGF-supplemented serum-free mammosphere formation is a standard method for detecting the self-renewal ability of CSCs. RICH1 knockdown resulted in an increase in the size and number of both primary and secondary mammospheres in MCF10A. Consistently, RICH1 overexpression significantly inhibited tumorsphere formation in SUM159 and BT549 (Fig. 3E). For CSC properties-associated transcription factors (CSC-TFs) OCT4, SOX2 and NANOG, we examined the mRNA expression levels of above mentioned CSC-TFs and found that RICH1 knockdown upregulated CSC-TFs in MCF10A, whereas RICH1 overexpression suppressed CSC-TFs in BT549 and SUM159 (Fig. 3F, G).

According to the study by Wells et al. [13], RICH1 plays an important role in maintaining the apical-basal polarity of MDCK epithelial cells by its interaction with Amot. To further gain insight into the role of RICH1 in epithelial cell polarity maintenance, we performed a 3D morphogenesis experiment using MCF10A cells. After culturing for 15 days, the control group of MCF10A formed spherical acini with centrally apoptotic cells located, while more than 40% of RICH1 knockdown MCF10A cells formed aberrant acini structures, showing enlarged spheroid size or complex multiacinar structures without apoptosis of glandular lumen cells (Fig. 3H). Polarity was also assessed using the epithelial marker E-cadherin. E-cadherin staining in 3D morphogenesis assays showed mislocalization and discontinuous distribution of E-cadherin in RICH1-knockdown MCF10A cells (Fig. 3I). These results suggested that RICH1 downregulation disrupted the epithelial organization of MCF10A cells.

### RICH1 inhibits the invasion and metastasis of breast cancer and increases the sensitivity to chemotherapy drugs

**Fig. 4:**
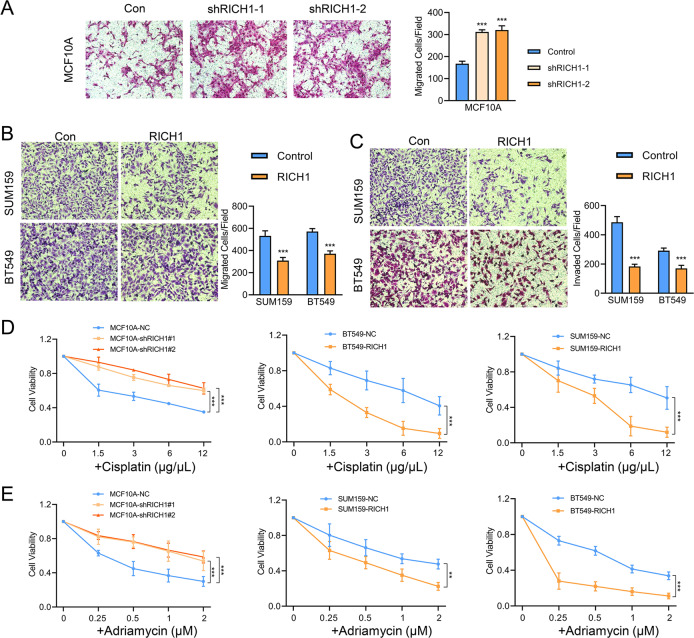
RICH1 inhibits the invasion and metastasis of breast cancer and increases the sensitivity to chemotherapy drugs. **A**–**C**. Cell migration and invasion assessed by transwell assay. The number of migrated or invaded cells were presented as the mean ± SD of three independent experiments. **D** CCK8 assays of NC- and RICH1-intervention cells after cisplatin (**D**) and adriamycin (**E**) treatment in various concentrations. All the experiments were repeated three times independently with similar results. Data represent mean ± SD. Statistical significance was determined by two-tailed unpaired *t*-test. Abbreviation: **p < 0.01; ***p < 0.001.

In addition to self-renewal capacity, other characteristics of CSCs include enhanced ability of invasion and metastasis and resistance to chemotherapy. We then performed transwell assays to test the effect of RICH1 on cell migration and invasion. The migration assays showed that the number of migrating cells in RICH1-knockdown groups was more than twice that of the control group (Fig. 4A), and overexpression of RICH1 would reduce about one-third migrating cells (Fig. 4B). The number of cells invading across the matrigel was also significantly decreased in RICH1 overexpression groups (Fig. 4C). When the cells were treated with cisplatin or adriamycin, we found that RICH1 knockdown MCF10A cells were more resistant than control cells to chemotherapeutic drugs, whereas RICH1 overexpression induced susceptibility to cisplatin and adriamycin in SUM159 and BT549 (Fig. 4D, E).

### RICH1 activates the kinases cascade of Hippo signaling and inhibits nuclear translocation of YAP/TAZ

**Fig. 5:**
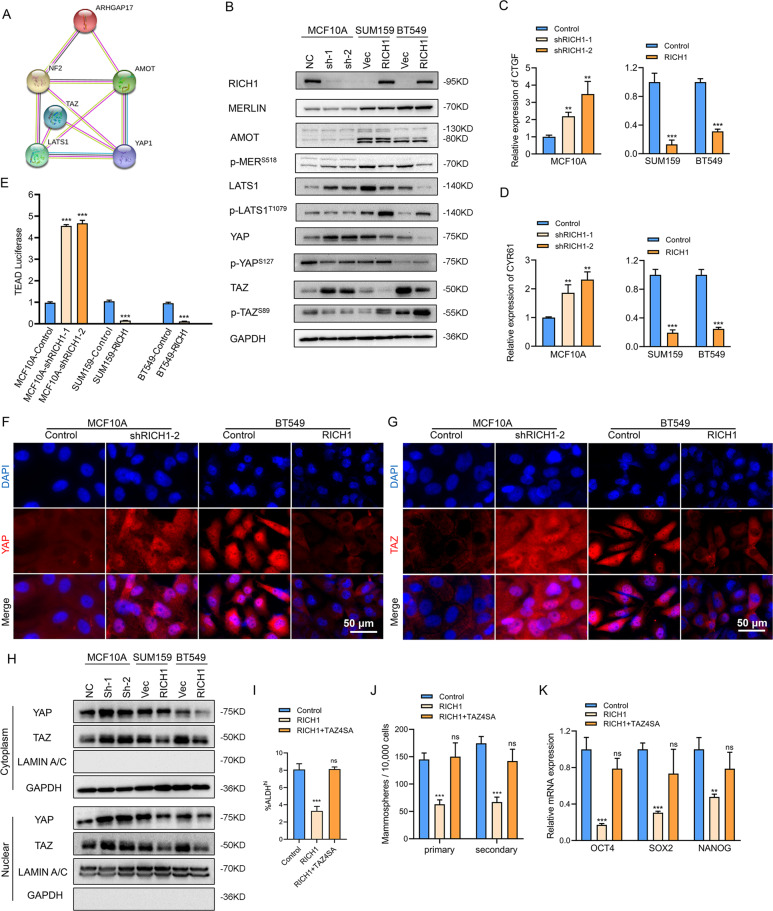
RICH1 activates the kinases cascade of Hippo signaling and inhibits nuclear translocation of YAP/TAZ. **A** The protein-protein interaction network between RICH1 and main regulators and effectors of Hippo signaling identified by STRING database. ARHGAP17-gene symbol for RICH1; NF2-gene symbol for MERLIN. **B** Detection of main regulators and effectors of Hippo signaling after RICH1 knockdown and overexpression by Western blotting. **C, D** The mRNA expression levels of YAP/TAZ target genes CTGF (C) and CYR61 (D) assayed by real time PCR. **E** TEAD-luciferase reporter analysis in RICH1-knockdown and -overexpressing cells. **F**, **G** Localization of YAP and TAZ shown by immunofluorescence assays. **H** Cytoplasmic and nuclear expression of YAP and TAZ determined by Western blotting. GAPDH was used as the loading control for the cytoplasmic protein. Lamin A/C was used as the loading control for nuclear protein. **I** Quantitation of ALDH^hi^ subpopulations in BT549 with RICH1 overexpression and TAZ4SA transduction. **J** Quantitation of primary and secondary tumorsphere formation in BT549 cells with RICH1 overexpression and TAZ4SA transduction. **K** Relative mRNA expression levels of CSC-TFs (OCT4, SOX2, and NANOG) in BT549 cells with RICH1 overexpression and TAZ4SA transduction. All the experiments were repeated three times independently with similar results. Data represent mean ± SD. Statistical significance was determined by two-tailed unpaired *t*-test. Abbreviation: ns, not significant; **p < 0.01; ***p < 0.001.

Since the close link between RICH1 and other upstream regulators of the Hippo signaling has been demonstrated in several previous studies [13, 20], and the core transcriptional coactivators YAP/TAZ of Hippo pathway have been identified as key regulators of CSCs-related traits on breast cancer cells, we further used the STRING database to predict the protein-protein interaction network of RICH1-associated molecules and found that RICH1 was involved in the regulatory network of Hippo signaling via interaction with Merlin or Amot (Fig. 5A). We then detected the protein expression levels of main regulators and effectors of Hippo signaling, to test whether RICH1 can indeed regulate the kinases cascade of the Hippo pathway. When knocking down RICH1 expression in MCF10A, the phosphorylation of Merlin in Ser518 was enhanced and the total LATS1 level increased. Consistently, the downstream total YAP/TAZ levels also increased, while the phosphorylation of YAP in Ser127 and TAZ in Ser89 were downregulated. In SUM159 and BT549, RICH1 overexpression inhibited the phosphorylation of Merlin^S518^, and promoted the phosphorylation of LATS1^T1079^, YAP^S127^, and TAZ^S89^, whereas the total protein levels of LATS1, YAP, and TAZ decreased significantly (Fig. 5B). Next, we determined whether RICH1 could modulate the expression of YAP/TAZ target genes, such as CTGF and CYR61. The results showed that the mRNA expressions of CTGF and CYR61 increased in RICH1 downregulated cells, and decreased in RICH1 overexpression cells (Fig. 5C, D). YAP/TAZ facilitates the transcription of downstream target genes by directly binding to the transcription factor TEADs. Then we monitored TEAD activity based on the 8×GTIIC luciferase reporter. We observed that RICH1 depletion increased TEAD luciferase activity in MCF10A, and RICH1 overexpression reduced the luciferase signals (Fig. 5E).

In addition to the protein expression levels of YAP/TAZ, the subcellular localization of YAP/TAZ is also crucial for the activity of the Hippo pathway. Immunofluorescence assays (Fig. 5F, G) and separation of cytoplasmic and nuclear protein extractions (Fig. 5H) were further performed to show that RICH1 knockdown in MCF10A could significantly promote the translocation of YAP/TAZ into the nucleus, while overexpression of RICH1 in BT549 could inhibit the cytonuclear shuttling of YAP/TAZ. Jointly, these data supported that RICH1 could facilitate the phosphorylation and cytoplasmic retention of YAP/TAZ, thus resulting in the blockage of YAP/TAZ-mediated transcription signal downstream of the Hippo pathway.

Since the transcriptional coactivator TAZ plays a more important role in the regulation of BCSC-like properties [6], we next tested whether the inhibition of BCSCs-like properties by RICH1 could be reversed by TAZ4SA, a mutant vector of TAZ with serine at all LATS-phosphorylation sites were mutated to alanine [22]. As expected, the introduction of TAZ4SA reversed the ALDH^hi^ subpopulation reduction in BT549 induced by RICH1 overexpression (Fig. 5I and Supplementary Fig. 2). Consistently, primary and secondary tumorsphere formation (Fig. 5J) and the expression levels of CSC-TFs (Fig. 5K) were also restored by TAS4SA in RICH1-overexpressing cells.

### RICH1 inhibits stem cell-like properties of breast cancer in vivo

**Fig. 6:**
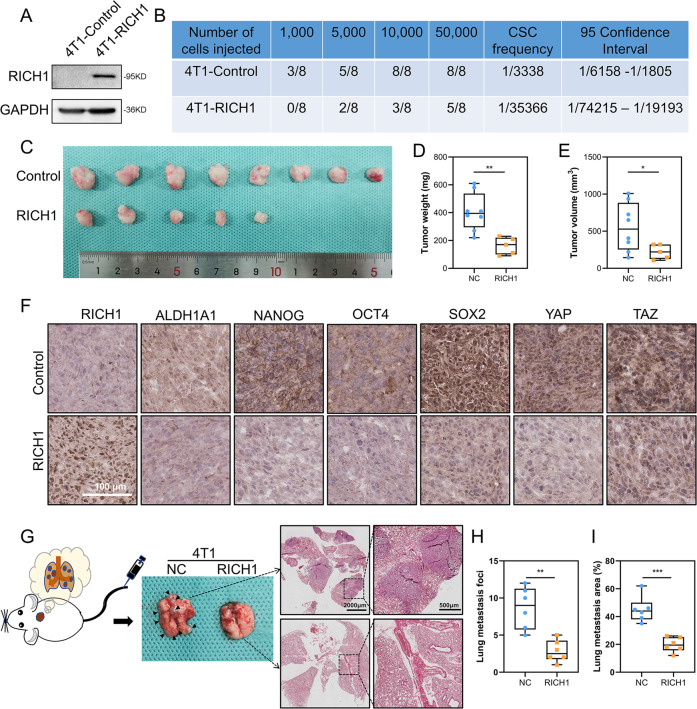
RICH1 inhibits stem cell-like properties of breast cancer in vivo. **A** RICH1 overexpression in 4T1 cells. **B** In vivo tumor formation of the mice injected with serial dilutions of 4T1-Control and 4T1-RICH1 cells. **C** Tumor images of mice injected with 50,000 control and RICH1-overexpressing 4T1 cells. **D, E**. Tumor weights (**D**) and volumes (**E**) of mice injected with 50,000 control and RICH1-overexpressing 4T1 cells. **F** Representative IHC staining results of tumor samples. **G** Representative visualized lung metastasis models and HE staining of lung metastasis specimen (arrows: metastatic foci). **H** Number of metastatic foci of control and RICH1-overexpressing 4T1 cells. **I** Lung metastasis area of control and RICH1-overexpressing 4T1 cells. Statistical significance was determined by two-tailed unpaired *t*-test. Abbreviation: *p < 0.05; **p < 0.01; ***p < 0.001.

To examine the role of RICH1 in tumorigenesis in mice, we overexpressed RICH1 in 4T1 (Fig. 6A), and 1×10^3^ - 5×10^4^ 4T1 control or RICH1-overexpressing cells were orthotopically transplanted into female Balb/c mice. When 5×10^4^ cells were injected, all 8 mice developed tumors in a control group, while only five of the eight mice developed tumors in the RICH1-overexpression group (Fig. 6B, C). When the fewer cells were inoculated, the weaker tumor-initiating capability was observed in the overexpression group. While 3/8 of the mice injected with 1×10^3^ control cells succumbed to tumors, none tumor was detected in the RICH1-overexpression group, with the CSC frequency decreasing to nearly 1/10 after RICH1 overexpression (Fig. 6B). Tumor weights and volumes were also obviously reduced in mice inoculated with RICH1-overexpressing cells (Fig. 6D, E). IHC analysis showed that overexpression of RICH1 significantly inhibited the expression of YAP/TAZ and CSCs-associated markers (ALDH1A1, NANOG, OCT4, and SOX2) in transplanted tumor tissues (Fig. 6F).

In addition to the enhanced capacity of tumorigenesis, CSCs are also regarded as one of the origins of distant metastasis. Therefore, the tail vein injection models were then used to verify the effect of RICH1 on the metastasis ability of tumor cells in vivo. We injected the control and RICH1-overexpressing 4T1 cells in Balb/c, and the number and area of lung metastases were obviously reduced in 4T1-RICH1 group (Fig. 6G–I). These results indicated that RICH1 could suppress the tumorigenesis and distant metastasis in vivo.

### RICH1 activates Hippo signaling by competing with Merlin for binding to Amot-p80

**Fig. 7:**
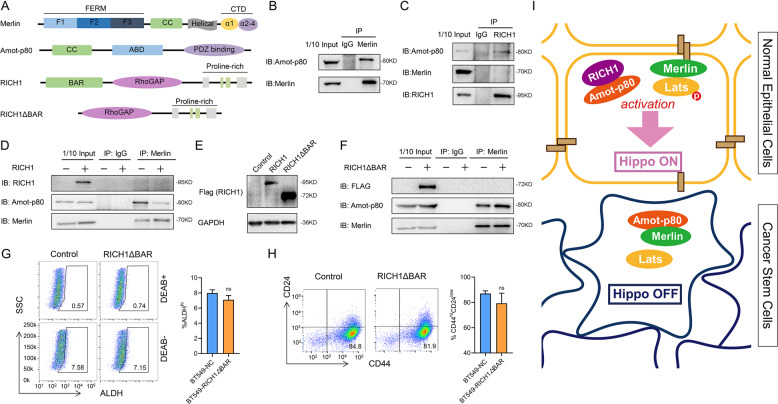
RICH1 activates Hippo signaling by competing with Merlin for binding to Amot-p80. **A** Domain organizations of Merlin, Amot-p80, RICH1 and RICH1△BAR. **B** Co-IP analysis of Merlin-Amot-p80 interaction in BT549 cells. **C** Co-IP analysis of interaction between RICH1, Amot-p80 and Merlin in RICH1-overexpressing BT549 cells. **D** Co-IP analysis of interaction between Merlin and Amot-p80 in control and RICH1-overexpressing BT549 cells. **E** Overexpression of RICH1 and RICH1△BAR (truncation mutant of RICH1 lacking the N-terminal BAR domain) in BT549 cells. **F** Co-IP analysis of interaction between Merlin and Amot-p80 in control and RICH1△BAR-overexpressing BT549 cells. **G, H** Flow cytometry analysis and representative images of ALDH^hi^ (**G**) and CD44^+^CD24^**−**^ (**H**) subpopulations in BT549 cells. Numbers in the flow cytometry charts indicate the CSC percentages. **I** Schematic model of the role of RICH1 in BCSC regulation. The experiments were repeated three times independently with similar results. Data represent mean ± SD. Statistical significance was determined by two-tailed unpaired *t*-test. Abbreviation: ns not significant.

Based on the protein-protein interaction network predicted by the STRING database, RICH1 has the potential to interact with Amot and Merlin. According to previous reports, Amot and Merlin could bind to each other by their mutual coiled-coiled (CC) motifs, and RICH1 could bind to the CC motif of Amot through the BAR domain in its N-terminal [13, 20]. Since the endogenous expression levels of Amot-p130 subtype were very scarce in the cell lines adopted in our study, the following mechanism research focused on the Amot-p80 subtype. We then verify the binding interaction between Merlin and Amot-p80 in BT549 using Co-IP (Fig. 7B). In BT549 with RICH1 exogenous overexpression, Amot-p80 was identified to bind with RICH, while Merlin could not (Fig. 7C). Next, we examined whether the exogeneous expression of RICH1 in BT549 could interfere the binding of Amot-p80 and Merlin. We found that the contents of Amot-p80 binding with Merlin was significantly decreased when RICH1 was overexpressed (Fig. 7D). Considering the BAR domain has been widely recognized as the binding site of CC motif, we then constructed truncation mutant of RICH1 (RICH1△BAR) lacking the N-terminal BAR domain (Fig. 7E). Deletion of the BAR domain of RICH1 abolished the function of inhibiting the binding of Amot-p80 and Merlin (Fig. 7F). Subsequently, we examined the effect of RICH1△BAR on the proportions of BCSC subpopulations, and no significant changes in the percentage of BCSCs populations were found in BT549 with RICH1△BAR exogenous expression (Fig. 7G, H). These results confirmed that RICH1 inhibited the BCSCs-like characteristics, by competing with Merlin for binding to Amot-p80 to activate the Hippo kinases cascade.

## Discussion

This study has identified the role of RICH1, a cell polarity-associated protein, in inhibiting the BCSCs-related traits, by activating the canonical Hippo signaling via displacing Amot-p80 from the complex with Merlin. Although the regulation of cytoskeletal remodeling in neurocytes and platelets by RICH1 are well defined [14–18], the exact role of RICH1 in oncogenesis and progression has been poorly determined. It was known that RICH1, as the downstream of VEGF/NRP1 signaling, was involved in the regulation of filopodia formation and migration of TNBC cells by modulating Cdc42 [19]. In schwannoma, the GTPase-activating efficacy of RICH1 was modulated by the Amot/Merlin complex, which subsequently inhibited cellular proliferation in vitro and tumorigenesis in vivo [20]. However, the tumor-suppressive function of RICH1 in the abovementioned studies, whether in inhibiting tumor cells migration or proliferation, was achieved through its catalyzing function of small Rho GTPases. In this study, we reported a previously unidentified role of RICH1 in stemness regulation of breast cancer. By comparing the CSCs and non-CSCs-like subpopulations separated from a TNBC cell line BT549, and analyzing the correlation between RICH1 and ALDH1A1 in TNBC samples, the negative correlation between RICH1 and CSC properties of breast cancer cells was firstly found in our study. We also demonstrated that RICH1 contributed to the dissociation of Amot-p80 and Merlin, leading to activation of the Hippo pathway and downregulation of YAP/TAZ for CSC regulation. Although the detailed mechanism of RICH1 to mediate the interaction of Amot-p80 and Merlin is yet to be determined, our study will expand our understanding of RhoGAP family proteins and CSC regulation in breast cancer.

The Hippo transducer YAP/TAZ is verified to sustain self-renewal and tumor-initiation capacities in BCSCs. Nuclear YAP could interact with β-catenin and TEAD4 at gene regulatory elements to promote basal-like breast cancer formation, luminal to basal trans-differentiation and the acquisition of CSCs-related properties [9]. In mammary epithelial cells, TAZ forms a complex with cell polarity protein Scribble, and the loss of Scribble leads to activation of TAZ by disrupting the inhibitory effects of core kinase Mst and Lats, thus endowing self-renewal capacity to non-CSCs [6]. The activity of YAP/TAZ is mainly modulated by the Hippo core kinase cascades, and the cell-polarity determinant proteins have been reported to regulate Hippo signaling by interacting with one component of Mst-Lats-YAP/TAZ axis [23, 24]. Although Merlin and Amot are well known as the Hippo kinases regulators [10, 12, 25–28], how upstream signals regulate the activity of Merlin and Amot remains a major open question. We firstly identified RICH1 could activate the kinases cascade of Hippo pathway by competing with Merlin for binding to Amot-p80, and blocking the phosphorylation of Merlin at Ser518 into an inactivated conformation. Previous studies about the conformation regulation of Merlin suggested that phosphorylation at Ser518 could prevent the binding of FERM domain and C-terminal tail, leaving Merlin in an “open” and inactive conformation [29, 30]. Considering the CC domains containing in Merlin and Amot are widely participated in the formation of homologous or heterologous polymers, we further validated whether the deletion of BAR domain in RICH1 could affect the binding of Amot-p80 and Merlin. As expected, the truncation mutant of RICH1 with deletion of BAR domain abolishes the inhibitory effect on the binding of Amot-p80 and Merlin. However, how the weakened combining of Amot-p80 and Merlin leads to the decreased phosphorylation of Merlin and the activation of downstream kinase cascades still needs to be further explored.

In addition to the well-established core components of Hippo signaling, other additional upstream regulators of YAP/TAZ activity have emerged, such as the transcriptional activation of YAP/TAZ by mechanotransduction driven through Rho-GTPase/F-actin signaling, a manner largely independent of MST and LATS1/2 [refs. 11, 22, 31]. For exogenous expression of Rich△BAR had no effect on the CSCs-like properties in breast cancer, and the deletion of BAR domain in RICH1 could not reduce the GTPase hydrolyzing function for Cdc42 (data not shown), we suggested that the potential RICH1-Cdc42-F-actin-YAP/TAZ regulatory axis was of little significance in the regulation of tumor stemness. However, whether the crosstalk between Rho and Hippo signaling functions in the downstream of RICH1, along with the biological significance of this interaction, are still need to be further explored.

Loss of cell polarity is a fundamental histopathological trait of most human cancers. The mechanistic relationship between loss of cell polarity and acquisition of CSC characteristics remains an important topic in cancer biology [32–34]. We found that loss of RICH1 in immortalized mammary epithelial cell MCF10A could promote the acquisition of CSCs-like properties, accompanied by the disorder of cell polarity. The present study links the CSC concept to the Hippo signaling by revealing a mechanistic basis of the regulation of Hippo kinase cascade by cell polarity proteins.

In conclusion, our results demonstrate that the low expression of RICH1 is associated with poor prognosis and CSCs-like properties in breast cancer, and its overexpression potentiates Hippo signaling through competing with Merlin for binding to Amot-p80, which further inhibits stemness and improve the chemosensitivity of breast cancer cells. Our work elucidates the role and molecular mechanism of RICH1 in tailoring BCSC-like characteristics, providing a new avenue to diagnosis and treatment of breast cancer.

## Materials and methods

### Patients and datasets

The mRNA expression levels of RICH1 in 1217 samples, including1104 breast cancer samples and 113 normal breast samples, and the corresponding clinical information were downloaded from the TCGA database (http://portal.gdc.cancer.gov). The 12 pairs of breast cancer tissues and adjacent non-cancerous tissues for RNA isolation were collected from patients who were diagnosed with breast cancer at the First Affiliated Hospital of Xi’an Jiaotong University (Shaanxi, China). All the patients had signed the informed consent before the surgery, and didn’t suffer from other malignancy or received radiotherapy or chemotherapy. Tissues were obtained after surgical excision and stored immediately in liquid nitrogen for subsequent use. One hundred and forty breast cancer tissues and 77 paired adjacent noncancerous tissues for immunohistochemistry (IHC) staining were obtained from the commercial tissue microarrays (HBreD140Su03 and HBreD077Su01, Outdo Biotech, Shanghai, China). A total of 135 tumor samples and 57 adjacent mammary gland tissues were included in the final analysis after the removal of dropping samples and patients without complete clinical information. Kaplan-Meier survival analysis was subsequently performed on 134 breast cancer patients with complete RFS and OS follow-up information. RNA-seq data of primary breast cancer and cancer mammospheres were obtained from GSE7515 [21] (https://www.ncbi.nlm.nih.gov/geo). The method for mammosphere growth from human breast cancer tissue samples had been described in detail in Creightons’ study [21]. In summary, core biopsies were placed immediately in cold RPMI-1640 supplemented with 10% heat-inactivated newborn calf serum, and then digested in collagenase. Subsequently, these cells were resuspended and used in mammosphere assays by plating these cells onto nonadherent (polyhema-coated) plastic, counting with a hemocytometer, and seeding 20,000 cells into 6-well ultralow attachment plates. RNA was then purified from cancer-derived mammospheres and primary breast cancer for use in gene expression profiling.

The present study was authorized by the Ethics Committee of the First Affiliated Hospital of Xi’an Jiaotong University and conducted in conformity to the Declaration of Helsinki.

### Cell culture and reagents

MCF10A, BT549, and 4T1 cells were purchased from Beijing union medical college hospital cell resource-sharing platform. SUM159 cells were a gift from Dr. Jianjun He. MCF10A was grown in DMEM/F12 media supplemented with 5% horse serum, 20 ng/ml human EGF, 10 μg/ml insulin, 0.5 μg/ml hydrocortisone, 1% penicillin and streptomycin. BT549 was cultured in RPMI 1640 medium with 10% fetal bovine serum (FBS), 1 μg/ml insulin, and 1% penicillin and streptomycin. SUM159 was cultured in DMEM/F12 medium with 10% FBS and 1% penicillin and streptomycin. 4T1 was cultured in RPMI 1640 medium with 10% FBS and 1% penicillin and streptomycin. Medium, FBS and Penicillin-Streptomycin were purchased from Corning. All these cell lines were maintained in a humidified incubator at 37°C, 5% CO_2_. Antibodies against RICH1 was purchased from Santa Cruz (sc-514438). Antibodies against Merlin (ab109244), ALDH1A1 (ab134188), Nanog (ab109250), Oct4 (ab181557), E-Cadherin (ab40772) were purchased from Abcam. Antibodies against LATS1 (#3477), phospho-LATS1^T1079^ (#8654), YAP (#14074), phosphor-YAP^S127^(#13008), TAZ (#70148), phosphor-TAZ^S89^(#59971),CD44 (#3570) were purchased from Cell Signaling Technology. The antibody against Amot was synthesized by Genemed Synthesis Inc. HRP-labeled GAPDH (HRP-60004) and antibody against lamin A/C (10298-1-AP) were from Proteintech.

### IHC

The paraffin-embedded tissue sections were roasted in 60 °C for 3 h and deparaffinized in xylene and dehydrated in gradient concentration of ethanol. Antigen repair of tissue slides was subsequently performed in sodium citrate buffer (PH = 9.0) in the microwave oven. The endogenous peroxidase was then deactivated by 3% hydrogen peroxide for 15 min. Subsequently, 5% BSA (bovine serum albumin) was applied to block nonspecific antigens at room temperature for 30 min and the incubation with primary antibodies in a certain concentration was performed overnight at 4 °C. On the second day, the slides were incubated with the homologous secondary antibody at room temperature for 1 h. Then, DAB (diaminobenzidine) staining, hematoxylin staining, dehydration in gradient ethanol, and transparency in xylene were followed to handle the slides. 10 random fields of each tissue section were selected for semi-quantitative scoring, and the scoring method was as follows: 1) Positive cell rate score – 0 for <10% positive cells, 1 for 10%~25% positive cells, 2 for 25%~50% positive cells, 3 for 50%~75% positive cells and 4 for >75% positive cells; 2) Dyeing strength score – 1 for light yellow, 2 for brown yellow and 3 for tan; 3) The total score was the product of the positive cell rate score and staining intensity score.

### Vector construction and cell transfection

To knock down RICH1, two shRNAs targeting RICH1 were obtained from Sigma website (https://www.sigmaaldrich.com/), and then used to construct shRNA plasmid vector by GeneChem. Then the shRNA against RICH1 and negative control shRNA-NC were packaged into lentivirus. The sequences of shRNAs targeting RICH1 were listed in Table 2. The overexpression vector containing the full-length or BAR domain deletion cDNA of RICH1 was synthesized and packaged into lentivirus by GeneChem. Cells were infected using transfection reagents HiTransG P (GeneChem) and cultured in the puromycin-containing medium for 2 weeks to generate stable RICH1 knockdown or overexpression cells.

**Table 2:** The sequences of shRNA and primers for real-time PCR assays.

Gene	Sequence
shRICH1#1	CCCAAGCAGATTACCATAGAA
shRICH1#2	GCCAGAGTTCTTCAGGAACAT
GAPDH-F	GGAGCGAGATCCCTCCAAAAT
GAPDH-R	GGCTGTTGTCATACTTCTCATGG
RICH1-F	CCTCTGTACGGCATAGCTGAG
RICH1-R	TGAGGATTTGTGAGCTTGGTTC
OCT4-F	CTTGAATCCCGAATGGAAAGGG
OCT4-R	GTGTATATCCCAGGGTGATCCTC
SOX2-F	GCCGAGTGGAAACTTTTGTCG
SOX2-R	GGCAGCGTGTACTTATCCTTCT
NANOG-F	TTTGTGGGCCTGAAGAAAACT
NANOG-R	AGGGCTGTCCTGAATAAGCAG
CTGF-F	AAAAGTGCATCCGTACTCCCA
CTGF-R	CCGTCGGTACATACTCCACAG
CYR61-F	CTCGCCTTAGTCGTCACCC
CYR61-R	CGCCGAAGTTGCATTCCAG

### RNA isolation and real-time PCR

Total RNA from tissue samples and cell lines was extracted using TRIzol reagent (Invitrogen), and then converted to cDNA using the PrimeScriptTM RT Master Mix (Takara) in accordance with the manufacture’s instructions. Real-time PCR was conducted with the SYBR-Green kit (Takara) to detect the mRNA expression levels. The primers used for real-time PCR were listed in Table 2 and purchased from Sangon Biotech.

### Western blotting

Whole-cell lysates were prepared by RIPA buffer, then proteins were separated with SDS/PAGE gel and transferred to PVDF membranes, which were incubated overnight with primary antibodies. Subsequently, HRP-conjugated secondary antibodies (Cell Signaling Technology) were incubated and chemiluminescent signals were detected by ECL (Millipore).

### Flow cytometry

Cells in the logarithmic growth phase were collected and resuspended with PBS. Then 500,000 cells were incubated with antibodies at recommended concentration at 37 °C for 30 min in dark according to the manufacturer’s instructions, followed by twice of PBS washing. After that, cells were filtered by a 70 μm strainer and sorted by a MoFlo XDP Flow Cytometer (Beckman) or analyzed by Canto II Flow Cytometer (BD). ALDH activity was detected by ALDHFLUOR Kit (StemCell Technologies), and cells treated with the specific ALDH inhibitor DEAB (diethylaminobenzaldehyde) was served as negative control. APC-conjugated CD44 antibody (#103012, Biolegend) and PE-conjugated CD24 antibody (#311106, Biolegend) were used to detect CD44^+^CD24^**−**^ subpopulations. Flow cytometry data were processed using FlowJo 10.6.2.

### Tumorsphere formation

Ten thousand cells were seeded in ultra-low attachment 6-well plate (#3471, Corning) and cultured in serum-free DMEM/F12 media (Gibco) supplemented with EGF (20 ng/ml, Peprotech) and B27 (2%, Invitrogen) for 1 or 2 weeks. The spheres larger than 80 μm in diameter were counted as the primary generation of tumorspheres. Then the initial tumorspheres were collected after filtering by 70 μm strainer and digested into single cells by trypsin. The culturing condition and counting method of the consecutive tumorspheres was the same for the initial tumorspheres.

### Migration and invasion assays

The transwell chambers for the invasion assays were coated with matrigel (1:6 dilution with serum-free medium). 200,000 cells in 200 μl serum-free medium were seeded in the upper chamber of transwell inserts, and 700 μl 10%FBS-containing medium was added to the lower chamber. The plates were placed in 5% CO_2_ at 37 °C for 24–72 h. Then the cells were fixed in 4% PFA (paraformaldehyde) for 10 mins and followed by 5% crystal violet staining for 20 mins. The migrated or invaded cells were subsequently photographed and counted.

### Chemoresistance assay

Three thousand cells were seeded in each well of 96-well plate. Chemotherapy agents in various concentration (cisplatin 1.5–12 μg/μl, adriamycin 0.25–2 μM) or solvent as negative control were added into the wells. Cells were cultured for 48 h, followed by CCK8 (cell counting kit-8, MedChemExpress) assays of cell viability. Ten μl of CCK8 solution was added into each well, and after incubation for 2 h, the absorbance was measured at 450 nm using a microplate reader.

### 3D morphogenesis

100 μl growth factor-reduced matrigel (Corning, #354230) was added to each well of a four-well chamber slide (Nunc 177437 LAB-TekTM) and incubated at 37 °C for 15 mins to solidify. Cells were resuspended in assay medium (DMEM/F12 with 2% horse serum, 0.5 μg/ml hydrocortisone, 10 μg/ml insulin and 1% penicillin/streptomycin), and then mixed with assay medium plaus (DMEM/F12 with 2% horse serum, 10 ng/ml EGF, 0.5 μg/ml hydrocortisone, 10 μg/ml insulin, 1% penicillin/streptomycin, and 4% matrigel) in a ratio of 1:1. 400 μl cell suspension was carefully seeded into the precoated chamber slide to generate the final concentration of 5,000 cells/well. The chamber slide was then transferred to the incubator and the medium (2.5% matrigel and 5 ng/ml EGF) was replaced every 4 days.

### Immunofluorescence

Cells were fixed with 4% paraformaldehyde, permeabilized with 0.2% Triton X-100, and blocked with 5% BSA. Cells were incubated overnight with specific primary antibody, followed by fluorescent-labeled secondary antibody (Proteintech). DAPI was then used for DNA staining. Image capture was performed by a Leica inverted fluorescence microscope.

### Luciferase reporter assay

Cells were plated at a concentration of 20,000 cells/well in 24-well plates. After overnight serum starvation, the cells were co-transfected with 0.5 μg TEAD firefly luciferase reporter (Addgene, #34615) together with 0.25 μg Renilla luciferase plasmid as a control using Lipofectamine 2000 (Invitrogen). Luciferase activity was then measured by Dual-Luciferase Reporter Assay (Promega) using a GloMax 96 Microplate Luminometer 48 h later. The TEAD firefly luciferase activity was normalized to the internal Renilla luciferase activity.

### Immunoprecipitation

Total protein was extracted by NP-40 lysis buffer (20 mM Tris-HCl, 150 mM NaCl, 0.5% NP-40, 20% glycerol) and then centrifuged at 12,000 rpm 4 °C for 20 min. Cell lysates were incubated with magnetic beads to prepare bead-antibody-antigen complexes using 4 μg antibody and 50 μl protein A/G magnetic beads (Thermo Fisher, 10007D). After the binding protein was obtained by washing with elution buffer, protein complexes were boiled and subjected to western blotting.

### In vivo tumor experiments

All animal studies in this study were conducted in accordance with the guidelines provided by the Animal Ethics Committee of Xi’an Jiaotong University. Five weeks female Balb/c mice were obtained from the Laboratory Animal Center of Xi’an Jiaotong University, and randomly divided into control and experimental groups according to the random number table method. For limited-dilution assays, cells were resuspended in PBS at a concentration of 1 × 10^6^ cells/mL and diluted to specific concentrations (*n* = 8 at each dilution concentration). Balb/c mice were inoculated with 4T1 cells at specific concentration into the fourth mammary fat pad on the right side. The tumor incidence was examined by palpation and surgical incision of the mammary glands at the end of the experiment. Tumor weights were measured and tumor volumes were calculated by the formula (a × b^2^)/2, where a is the major tumor axis and b is the minor tumor axis. For the lung metastasis model of tumor cells, 1 × 10^6^ cells suspended in 200 μl PBS were injected into Balb/c mice via tail vein (n = 6). After 2 weeks of injection, lung tissues were collected for H-E (hematoxylin-eosin) staining. The relative number of lung metastases nodules was counted.

### Statistical analysis

All data were analyzed using Graphpad Prism 8, and all experiments were repeated at least three times. Student’s two-sided *t*-test was used to compare the differences between two groups. Differences in survival between different groups were compared by Kaplan-Meier curves followed by log-rank test. The correlation between clinical characteristics and RICH1 expression levels in human breast cancer patients’ samples were analyzed using the *χ*^2^ test. *P* < 0.05 was considered as statistically significant.

## Supplementary information

Supplementary Information File

Supplementary Figure 1

Supplementary Figure 2

Author Contribution Form

Reproducibility checklist for CDDIS-21-2425R

## Data Availability

The datasets used and/or analyzed during the current study are available from the corresponding author on reasonable request.
