# Supplementary material for: RICH1 inhibits breast cancer stem cell traits through activating kinases cascade of Hippo signaling by competing with Merlin for binding to Amot-p80
Source: Cell Death Dis. 2022 Jan 21;13(1):71. doi: 10.1038/s41419-022-04516-2 (PMC8782888; doi:10.1038/s41419-022-04516-2)
Supplement: Supplementary file 1 — Supplementary Information File [file 41419_2022_4516_MOESM1_ESM.docx]

**Supplementary Figure Legends**

**Supplementary Figure 1.** Representative images of flow cytometry results of CD44^+^CD24^-^ subpopulations in MCF10A (A), SUM159 (B) and BT549 (B) cells. **Supplementary Figure 2.** Representative images of flow cytometry results of ALDH^hi^ subpopulations in BT549 cells with RICH1 overexpression and TAZ4SA transduction.
