# Supplementary figures and images for: RICH1 inhibits breast cancer stem cell traits through activating kinases cascade of Hippo signaling by competing with Merlin for binding to Amot-p80
Source: Cell Death Dis. 2022 Jan 21;13(1):71. doi: 10.1038/s41419-022-04516-2 (PMC8782888; doi:10.1038/s41419-022-04516-2)

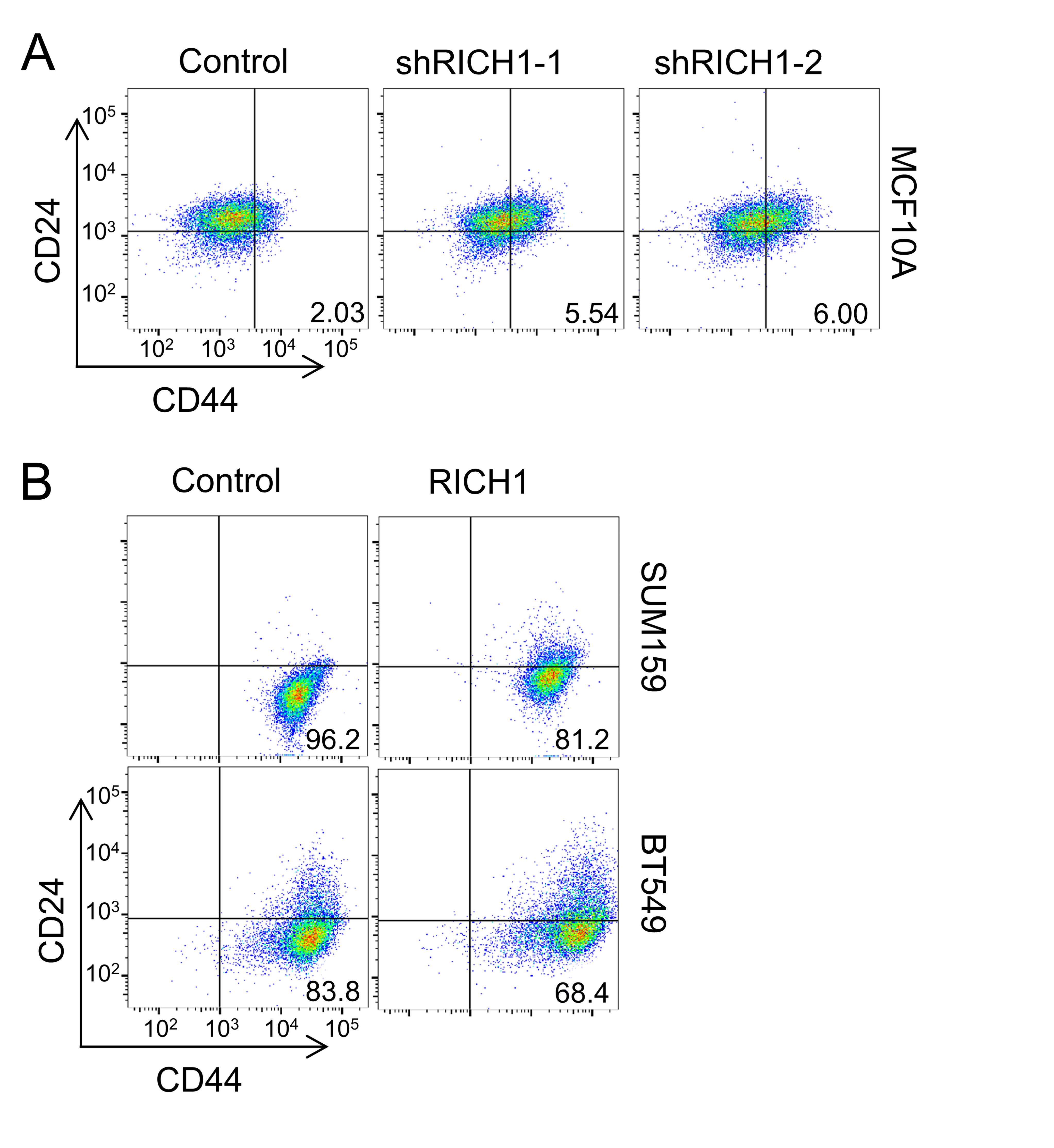

Supplement: Supplementary file 2 — Supplementary Figure 1 [file 41419_2022_4516_MOESM2_ESM.tif]

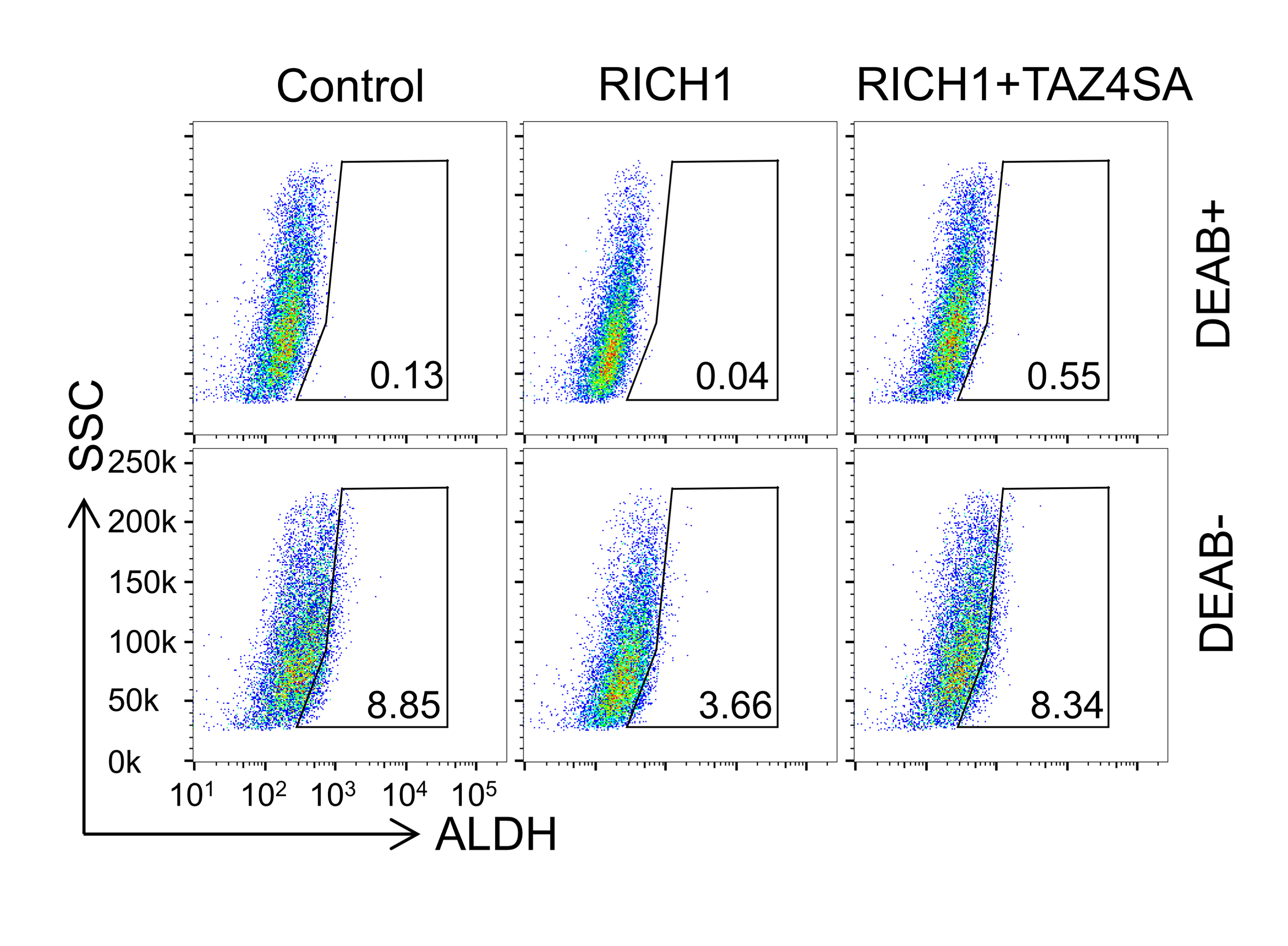

Supplement: Supplementary file 3 — Supplementary Figure 2 [file 41419_2022_4516_MOESM3_ESM.tif]
