# Supplementary material for: RICH1 inhibits breast cancer stem cell traits through activating kinases cascade of Hippo signaling by competing with Merlin for binding to Amot-p80
Source: Cell Death Dis. 2022 Jan 21;13(1):71. doi: 10.1038/s41419-022-04516-2 (PMC8782888; doi:10.1038/s41419-022-04516-2)
Supplement: Supplementary file 4 — Author Contribution Form [file 41419_2022_4516_MOESM4_ESM.pdf]

DECLARATION OF CONTRIBUTIONS TO ARTICLE

ADMC

Manuscript Number: CDDIS-21-2425

Journal Name: *Cell Death & Disease* (the 'Journal')

Proposed Title of the Contribution:

RICH1 inhibits breast cancer stem cell traits through activating kinases cascade of Hippo signaling via attenuating the binding of Amot-p80 and Merlin (the 'Contribution)

Author(s):

Qi Tian, Huan Gao, Yan Zhou, Lizhe Zhu, Jiao Yang, Bo Wang, Peijun Liu, Jin Yang (the 'Authors')

For all *CDDis* articles, each person named as an author in the published version must be able to show he or she has contributed substantially to the article.

Authorship credit should be based on 1) substantial contributions to conception and design, acquisition of data, or analysis and interpretation of data; 2) drafting the article or revising it critically for important intellectual content; and 3) final approval of the version to be published. Authors should meet conditions 1, 2 and 3.

Any person who cannot be shown to have made a substantial contribution to the article cannot be listed as an author in the final version. The name of any person who is deemed to have made a minor contribution can, however, appear in the Acknowledgments section of the article.

Please complete the table below to indicate the contributions of all named authors to the manuscript.

Author Full Name:	Specification of Contribution to the Manuscript:
Qi Tian	conceived the project, conducted all experiments, constructed the manuscript
Huan Gao	clinical sample collection
Yan Zhou	analyzed the data
Lizhe Zhu	clinical sample collection
Jiao Yang	analyzed the data
Bo Wang	offered experimental techniques support
Peijun Liu	supervised all experiments
Jin Yang	constructed the manuscript

Please complete the table below to indicate the contributions of all named authors to the figures.

Figure 1:

QT generated the data and assembled the figure. HG and LZZ collected the clinical specimens and performed PCR analyses. YZ performed survival analyses.

Figure 2:

BW supported the FACS techniques, LZZ collected the clinical specimens, JY performed the correlation analysis. QT analyzed all the data and assembled the figure.

Figure 3:

QT generated the data and assembled the figure. BW supported the 3D culture technique.

Figure 4:

QT generated the data and assembled the figure. JY guided the concentration range of chemo therapy drugs.

Figure 5:

QT generated the data and assembled the figure. PJL guided the TEAD luciferase reporter assay. JY guided the cytoplasma-nuclear extraction technique.

Figure 6:

QT generated the data and assembled the figure. LZZ supported in vivo experiment.

Figure 7:

QT generated the data and assembled the figure. BW helped the construction of truncation.

Signed for and on behalf of the Author(s):	Print Name:	Date:
Jin Yang	Jin Yang	2021/06/22
